# 2-(2-{[4-Oxo-3-(2-phenyl­eth­yl)-3,4-dihydro­quinazolin-2-yl]sulfan­yl}eth­yl)-2,3-dihydro-1*H*-isoindole-1,3-dione

**DOI:** 10.1107/S1600536812025883

**Published:** 2012-06-13

**Authors:** Adel S. El-Azab, Alaa A.-M. Abdel-Aziz, Abdulrahman M. Al-Obaid, Seik Weng Ng, Edward R. T. Tiekink

**Affiliations:** a Department of Pharmaceutical Chemistry, College of Pharmacy, King Saud University, Riyadh 11451, Saudi Arabia; b Department of Organic Chemistry, Faculty of Pharmacy, Al-Azhar University, Cairo 11884, Egypt; c Department of Medicinal Chemistry, Faculty of Pharmacy, University of Mansoura, Mansoura 35516, Egypt; d Department of Chemistry, University of Malaya, 50603 Kuala Lumpur, Malaysia; e Chemistry Department, Faculty of Science, King Abdulaziz University, PO Box 80203 Jeddah, Saudi Arabia

## Abstract

In the title compound, C_26_H_21_N_3_O_3_S, the quinazolinyl group is essentially planar [r.m.s. deviation for the 10 non-H atoms = 0.057 Å]. The isoindoline-1,3-dione group is linked by an SCH_2_CH_2_ chain to the pyrimidinyl C atom that lies between the two N atoms. Also, the phenyl group is linked by a CH_2_CH_2_ chain at the N atom adjacent to the carbonyl group. This results in a conformation with these substituents lying to either side of the central quinazolinyl unit, with the former being approximately parallel [dihedral angle = 4.93 (7)°], and the phenyl group being inclined [dihedral angle = 71.61 (9)°] to the central quinazolinyl fused-ring system. In the crystal, mol­ecules are consolidated into a three-dimensional architecture by C—H⋯O inter­actions, involving all three carbonyl-O atoms, and π–π inter­actions occurring between the pyrimidinyl and isoindole-benzene rings [inter-centroid distance = 3.5330 (13) Å].

## Related literature

For the synthesis and drug discovery trials of quinazoline-4(3*H*)-one derivatives, see: El-Azab & ElTahir (2012 ▶); El-Azab *et al.* (2011 ▶). For the synthesis and anti­microbial activity of the title compound, see: El-Azab (2007 ▶). For a related structure, see: El-Emam *et al.* (2012 ▶).
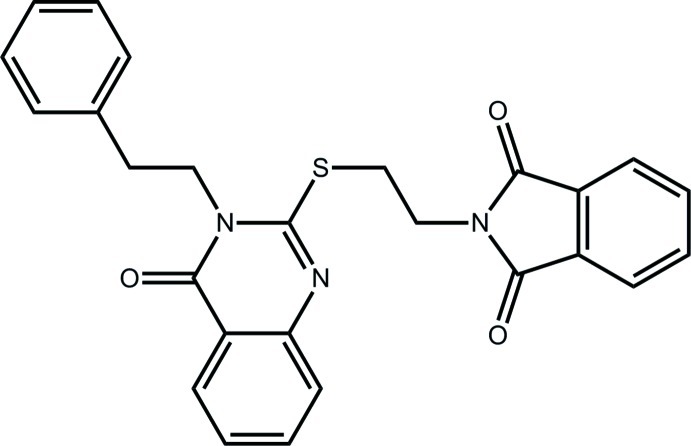


## Experimental

### 

#### Crystal data

C_26_H_21_N_3_O_3_S
*M* *_r_* = 455.52
Triclinic, 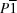

*a* = 8.7346 (4) Å
*b* = 9.4464 (6) Å
*c* = 13.7373 (8) Å
α = 94.258 (5)°
β = 103.505 (5)°
γ = 105.227 (5)°
*V* = 1052.27 (10) Å^3^
*Z* = 2
Cu *K*α radiation
μ = 1.66 mm^−1^
*T* = 100 K
0.25 × 0.15 × 0.02 mm

#### Data collection

Agilent SuperNova Dual diffractometer with Atlas detector
Absorption correction: multi-scan (*CrysAlis PRO*; Agilent, 2012 ▶) *T*_min_ = 0.511, *T*_max_ = 1.000
7763 measured reflections
4329 independent reflections
3482 reflections with *I* > 2σ(*I*)
*R*_int_ = 0.042

#### Refinement

*R*[*F* ^2^ > 2σ(*F* ^2^)] = 0.049
*wR*(*F* ^2^) = 0.139
*S* = 1.03
4329 reflections
298 parameters
H-atom parameters constrained
Δρ_max_ = 0.34 e Å^−3^
Δρ_min_ = −0.36 e Å^−3^

### 

Data collection: *CrysAlis PRO* (Agilent, 2012 ▶); cell refinement: *CrysAlis PRO*; data reduction: *CrysAlis PRO*; program(s) used to solve structure: *SHELXS97* (Sheldrick, 2008 ▶); program(s) used to refine structure: *SHELXL97* (Sheldrick, 2008 ▶); molecular graphics: *ORTEP-3* (Farrugia, 1997 ▶) and *DIAMOND* (Brandenburg, 2006 ▶); software used to prepare material for publication: *publCIF* (Westrip, 2010 ▶).

## Supplementary Material

Crystal structure: contains datablock(s) general, I. DOI: 10.1107/S1600536812025883/sj5240sup1.cif

Structure factors: contains datablock(s) I. DOI: 10.1107/S1600536812025883/sj5240Isup2.hkl

Supplementary material file. DOI: 10.1107/S1600536812025883/sj5240Isup3.cml

Additional supplementary materials:  crystallographic information; 3D view; checkCIF report

## Figures and Tables

**Table 1:** Hydrogen-bond geometry (Å, °)

*D*—H⋯*A*	*D*—H	H⋯*A*	*D*⋯*A*	*D*—H⋯*A*
C13—H13⋯O1^i^	0.95	2.56	3.218 (3)	127
C17—H17*B*⋯O3^ii^	0.99	2.42	3.120 (2)	128
C21—H21⋯O2^iii^	0.95	2.45	3.346 (3)	157

Symmetry codes: (i) 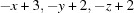; (ii) 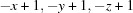; (iii) 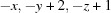.

**Fig. 1.**
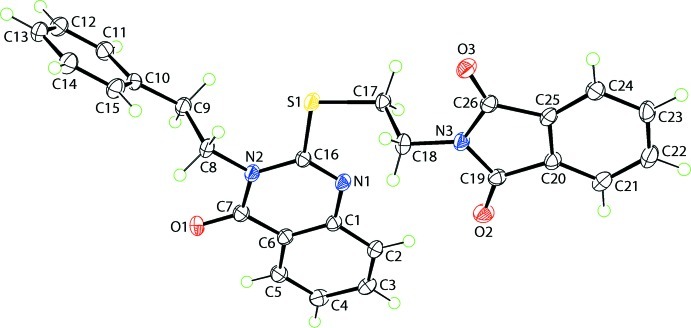
The molecular structure of (I) showing the atom-labelling scheme and displacement ellipsoids at the 50% probability level.

**Fig. 2.**
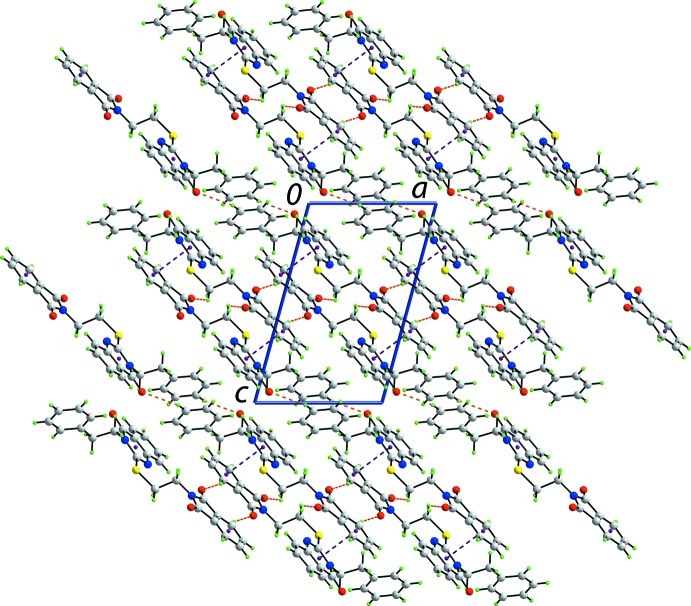
A view in projection down the b axis of the unit-cell contents for (I). The C—H···O and π—π contacts are shown as orange and purple dashed lines respectively.

## supplementary crystallographic information

### Comment

The title compound, 2-(2-(4-oxo-3-phenethyl-3,4-dihydroquinazolin-2-ylthio)ethyl)isoindoline-1,3-dione (I), was originally synthesized for evaluation of its anti-microbial activity (El-Azab, 2007) owing to the known biological activity of related quinazoline-4(3*H*)one derivatives (El-Azab & ElTahir, 2012; El-Azab *et al.*, 2011). Herein, we describe the crystal structure determination of (I).

In (I), Fig. 1, the quinazolinyl group is planar with the r.m.s. deviation for the 10 non-hydrogen atoms = 0.057 Å and maximum deviations of 0.062 (2) for the C5 atom and -0.068 (2) for the C7 atom. The isoindole (r.m.s. deviation for the nine non-hydrogen atoms = 0.018 Å), being linked by a SCH_2_CH_2_ chain at the C16 atom, and phenyl, linked by a CH_2_CH_2_ chain at the N2 atom, groups lie to either side of the molecule with the former being approximately parallel, dihedral angle = 4.93 (7)°, and the phenyl group being inclined, dihedral angle = 71.61 (9)°, with respect to the central quinazolinyl group.

Molecules are consolidated into a three-dimensional architecture by C—H···O interactions involving all three carbonyl-O atoms, Table 1, and π—π interactions occurring between the pyrimidinyl and isoindole-benzene rings [intercentroid distance = 3.5330 (13) Å, angle of inclination = 6.19 (10)° for symmetry operation: 1 - *x*, 2 - *y*, 1 - *z*], Fig. 2.

### Experimental

A mixture of 2-mercapto-3-phenethylquinazolin-4(3*H*)-one (564 mg, 2 mmol) and 2-(2-chloroethyl)isoindoline-1,3-dione (418 mg, 2.0 mmol) in acetone (10 ml) containing anhydrous K_2_CO_3_ (300 mg) was stirred at room temperature for 12 h. The reaction mixture was filtered, the solvent removed under reduced pressure and the solid obtained was dried and recrystallized from ethanol. Yield 89%; ^1^H NMR (CDCl_3_): δ = 8.10 (d, 1H, J = 7.5 Hz), 7.72 (dd, 2H, J = 3.0 Hz), 7.62–7.56 (m, 4H), 7.29 (t, 1H, J = 6.5, 7.0 Hz), 7.21–7.15 (m, 5H), 4.17 (t, 2H, J = 8.0, 8.5 Hz), 4.10 (t, 2H, J = 6.0, 6.5 Hz), 3.54 (t, 2H, J = 6.5 Hz), 2.94 (t, 2H, J = 8.0, 8.5 Hz) p.p.m.. MS (70 eV): m/*z* = 455.

### Refinement

Carbon-bound H-atoms were placed in calculated positions [C—H = 0.95 to 0.99 Å, *U*_iso_(H) = 1.2*U*_eq_(C)] and were included in the refinement in the riding model approximation.

### Crystal data

**Table:**

C_26_H_21_N_3_O_3_S	*Z* = 2
*M**_r_* = 455.52	*F*(000) = 476
Triclinic, *P*1	*D*_x_ = 1.438 Mg m^−^^3^
Hall symbol: -P 1	Cu *K*α radiation, λ = 1.54184 Å
*a* = 8.7346 (4) Å	Cell parameters from 2392 reflections
*b* = 9.4464 (6) Å	θ = 3.3–76.4°
*c* = 13.7373 (8) Å	µ = 1.66 mm^−^^1^
α = 94.258 (5)°	*T* = 100 K
β = 103.505 (5)°	Prism, colourless
γ = 105.227 (5)°	0.25 × 0.15 × 0.02 mm
*V* = 1052.27 (10) Å^3^	

### Data collection

**Table:**

Agilent SuperNova Dual diffractometer with Atlas detector	4329 independent reflections
Radiation source: SuperNova (Cu) X-ray Source	3482 reflections with *I* > 2σ(*I*)
Mirror monochromator	*R*_int_ = 0.042
Detector resolution: 10.4041 pixels mm^-1^	θ_max_ = 76.6°, θ_min_ = 3.3°
ω scan	*h* = −8→10
Absorption correction: multi-scan (*CrysAlis PRO*; Agilent, 2012)	*k* = −10→11
*T*_min_ = 0.511, *T*_max_ = 1.000	*l* = −17→14
7763 measured reflections	

### Refinement

**Table:**

Refinement on *F*^2^	Primary atom site location: structure-invariant direct methods
Least-squares matrix: full	Secondary atom site location: difference Fourier map
*R*[*F*^2^ > 2σ(*F*^2^)] = 0.049	Hydrogen site location: inferred from neighbouring sites
*wR*(*F*^2^) = 0.139	H-atom parameters constrained
*S* = 1.03	*w* = 1/[σ^2^(*F*_o_^2^) + (0.0728*P*)^2^ + 0.0527*P*] where *P* = (*F*_o_^2^ + 2*F*_c_^2^)/3
4329 reflections	(Δ/σ)_max_ = 0.001
298 parameters	Δρ_max_ = 0.34 e Å^−^^3^
0 restraints	Δρ_min_ = −0.36 e Å^−^^3^

### Fractional atomic coordinates and isotropic or equivalent isotropic displacement parameters (Å^2^)

**Table:**

	*x*	*y*	*z*	*U*_iso_*/*U*_eq_	
S1	0.78761 (6)	0.81994 (5)	0.66367 (4)	0.02236 (15)	
N3	0.2950 (2)	0.71976 (19)	0.53513 (14)	0.0199 (4)	
O1	1.09076 (18)	1.22980 (17)	0.94479 (12)	0.0258 (3)	
O2	0.23072 (19)	0.93889 (16)	0.56428 (13)	0.0268 (4)	
O3	0.27774 (18)	0.47861 (16)	0.47700 (12)	0.0238 (3)	
N1	0.7076 (2)	1.06895 (19)	0.69243 (13)	0.0191 (4)	
N2	0.9393 (2)	1.04543 (19)	0.81447 (13)	0.0193 (4)	
C1	0.7348 (2)	1.2099 (2)	0.74263 (16)	0.0187 (4)	
C2	0.6228 (3)	1.2899 (2)	0.70762 (17)	0.0220 (4)	
H2	0.5276	1.2453	0.6535	0.026*	
C3	0.6521 (3)	1.4336 (2)	0.75257 (17)	0.0231 (4)	
H3	0.5762	1.4873	0.7291	0.028*	
C4	0.7927 (3)	1.5015 (2)	0.83247 (17)	0.0240 (4)	
H4	0.8130	1.6012	0.8617	0.029*	
C5	0.9006 (3)	1.4224 (2)	0.86799 (17)	0.0225 (4)	
H5	0.9956	1.4675	0.9222	0.027*	
C6	0.8709 (2)	1.2757 (2)	0.82464 (16)	0.0199 (4)	
C7	0.9776 (2)	1.1879 (2)	0.86754 (16)	0.0210 (4)	
C8	1.0395 (2)	0.9495 (2)	0.85786 (16)	0.0210 (4)	
H8A	0.9743	0.8443	0.8360	0.025*	
H8B	1.0658	0.9687	0.9326	0.025*	
C9	1.1989 (3)	0.9758 (2)	0.82556 (17)	0.0216 (4)	
H9A	1.1719	0.9505	0.7511	0.026*	
H9B	1.2601	1.0825	0.8433	0.026*	
C10	1.3087 (2)	0.8866 (2)	0.87393 (15)	0.0193 (4)	
C11	1.4700 (3)	0.9199 (2)	0.86494 (17)	0.0239 (4)	
H11	1.5078	0.9981	0.8289	0.029*	
C12	1.5760 (3)	0.8415 (3)	0.90749 (17)	0.0264 (5)	
H12	1.6852	0.8665	0.9005	0.032*	
C13	1.5237 (3)	0.7268 (2)	0.96019 (17)	0.0253 (4)	
H13	1.5960	0.6723	0.9889	0.030*	
C14	1.3643 (3)	0.6925 (3)	0.97044 (18)	0.0265 (5)	
H14	1.3273	0.6142	1.0066	0.032*	
C15	1.2582 (3)	0.7718 (2)	0.92815 (17)	0.0240 (4)	
H15	1.1497	0.7474	0.9363	0.029*	
C16	0.8090 (2)	0.9957 (2)	0.72786 (16)	0.0190 (4)	
C17	0.5972 (2)	0.7934 (2)	0.56824 (16)	0.0208 (4)	
H17A	0.5999	0.8856	0.5379	0.025*	
H17B	0.5854	0.7134	0.5138	0.025*	
C18	0.4493 (2)	0.7530 (2)	0.61288 (16)	0.0223 (4)	
H18A	0.4520	0.6657	0.6482	0.027*	
H18B	0.4565	0.8366	0.6633	0.027*	
C19	0.1975 (2)	0.8162 (2)	0.51785 (16)	0.0199 (4)	
C20	0.0496 (2)	0.7337 (2)	0.43459 (16)	0.0194 (4)	
C21	−0.0828 (3)	0.7789 (3)	0.38598 (18)	0.0249 (5)	
H21	−0.0921	0.8745	0.4047	0.030*	
C22	−0.2021 (3)	0.6779 (3)	0.30825 (18)	0.0275 (5)	
H22	−0.2941	0.7058	0.2724	0.033*	
C23	−0.1893 (3)	0.5362 (3)	0.28182 (17)	0.0256 (5)	
H23	−0.2731	0.4693	0.2290	0.031*	
C24	−0.0544 (3)	0.4919 (2)	0.33240 (17)	0.0227 (4)	
H24	−0.0445	0.3960	0.3152	0.027*	
C25	0.0634 (2)	0.5941 (2)	0.40831 (16)	0.0199 (4)	
C26	0.2207 (2)	0.5824 (2)	0.47360 (16)	0.0196 (4)	

### Atomic displacement parameters (Å^2^)

**Table:**

	*U*^11^	*U*^22^	*U*^33^	*U*^12^	*U*^13^	*U*^23^
S1	0.0155 (3)	0.0206 (2)	0.0278 (3)	0.00622 (19)	0.00023 (19)	−0.00159 (19)
N3	0.0141 (8)	0.0202 (8)	0.0234 (9)	0.0042 (7)	0.0026 (7)	0.0012 (7)
O1	0.0174 (7)	0.0328 (8)	0.0219 (8)	0.0062 (6)	−0.0023 (6)	−0.0011 (6)
O2	0.0250 (8)	0.0216 (7)	0.0323 (9)	0.0062 (6)	0.0072 (7)	−0.0019 (6)
O3	0.0202 (7)	0.0214 (7)	0.0307 (8)	0.0087 (6)	0.0060 (6)	0.0026 (6)
N1	0.0127 (8)	0.0203 (8)	0.0227 (9)	0.0034 (6)	0.0036 (7)	0.0021 (7)
N2	0.0143 (8)	0.0223 (8)	0.0208 (9)	0.0058 (7)	0.0030 (7)	0.0032 (7)
C1	0.0137 (9)	0.0221 (9)	0.0204 (10)	0.0040 (7)	0.0060 (8)	0.0035 (8)
C2	0.0153 (10)	0.0258 (10)	0.0246 (11)	0.0060 (8)	0.0046 (8)	0.0024 (8)
C3	0.0195 (10)	0.0233 (10)	0.0296 (11)	0.0074 (8)	0.0100 (9)	0.0058 (8)
C4	0.0217 (10)	0.0193 (9)	0.0299 (11)	0.0028 (8)	0.0094 (9)	−0.0006 (8)
C5	0.0187 (10)	0.0246 (10)	0.0225 (10)	0.0031 (8)	0.0061 (8)	0.0018 (8)
C6	0.0152 (9)	0.0235 (10)	0.0211 (10)	0.0047 (8)	0.0063 (8)	0.0032 (8)
C7	0.0137 (9)	0.0256 (10)	0.0220 (10)	0.0029 (8)	0.0051 (8)	0.0015 (8)
C8	0.0143 (9)	0.0256 (10)	0.0232 (10)	0.0079 (8)	0.0018 (8)	0.0048 (8)
C9	0.0172 (10)	0.0257 (10)	0.0223 (10)	0.0075 (8)	0.0042 (8)	0.0048 (8)
C10	0.0150 (9)	0.0228 (9)	0.0179 (10)	0.0049 (7)	0.0018 (7)	0.0000 (8)
C11	0.0181 (10)	0.0303 (11)	0.0229 (11)	0.0053 (8)	0.0063 (8)	0.0038 (8)
C12	0.0152 (10)	0.0356 (12)	0.0285 (12)	0.0078 (9)	0.0061 (8)	0.0019 (9)
C13	0.0191 (10)	0.0288 (10)	0.0263 (11)	0.0096 (8)	0.0011 (8)	0.0007 (9)
C14	0.0220 (11)	0.0288 (11)	0.0300 (12)	0.0086 (9)	0.0071 (9)	0.0067 (9)
C15	0.0153 (10)	0.0284 (10)	0.0290 (11)	0.0060 (8)	0.0073 (8)	0.0052 (9)
C16	0.0141 (9)	0.0221 (9)	0.0191 (10)	0.0038 (7)	0.0037 (8)	0.0015 (8)
C17	0.0147 (9)	0.0202 (9)	0.0237 (10)	0.0038 (8)	0.0004 (8)	−0.0005 (8)
C18	0.0154 (10)	0.0253 (10)	0.0234 (11)	0.0043 (8)	0.0011 (8)	0.0036 (8)
C19	0.0179 (10)	0.0212 (9)	0.0221 (10)	0.0058 (8)	0.0078 (8)	0.0029 (8)
C20	0.0137 (9)	0.0222 (9)	0.0221 (10)	0.0036 (7)	0.0060 (8)	0.0036 (8)
C21	0.0210 (11)	0.0286 (10)	0.0300 (12)	0.0114 (9)	0.0101 (9)	0.0080 (9)
C22	0.0170 (10)	0.0390 (12)	0.0286 (12)	0.0103 (9)	0.0056 (9)	0.0112 (10)
C23	0.0139 (10)	0.0371 (12)	0.0217 (11)	0.0026 (9)	0.0030 (8)	0.0028 (9)
C24	0.0176 (10)	0.0255 (10)	0.0240 (11)	0.0035 (8)	0.0072 (8)	0.0014 (8)
C25	0.0148 (9)	0.0239 (10)	0.0236 (10)	0.0069 (8)	0.0077 (8)	0.0057 (8)
C26	0.0148 (10)	0.0216 (9)	0.0222 (10)	0.0037 (8)	0.0064 (8)	0.0019 (8)

### Geometric parameters (Å, º)

**Table:**

S1—C16	1.764 (2)	C9—H9B	0.9900
S1—C17	1.806 (2)	C10—C15	1.396 (3)
N3—C19	1.399 (3)	C10—C11	1.398 (3)
N3—C26	1.399 (3)	C11—C12	1.386 (3)
N3—C18	1.453 (3)	C11—H11	0.9500
O1—C7	1.226 (3)	C12—C13	1.385 (3)
O2—C19	1.209 (3)	C12—H12	0.9500
O3—C26	1.210 (3)	C13—C14	1.388 (3)
N1—C16	1.293 (3)	C13—H13	0.9500
N1—C1	1.388 (3)	C14—C15	1.391 (3)
N2—C16	1.391 (3)	C14—H14	0.9500
N2—C7	1.400 (3)	C15—H15	0.9500
N2—C8	1.481 (3)	C17—C18	1.528 (3)
C1—C6	1.397 (3)	C17—H17A	0.9900
C1—C2	1.408 (3)	C17—H17B	0.9900
C2—C3	1.381 (3)	C18—H18A	0.9900
C2—H2	0.9500	C18—H18B	0.9900
C3—C4	1.406 (3)	C19—C20	1.494 (3)
C3—H3	0.9500	C20—C21	1.380 (3)
C4—C5	1.376 (3)	C20—C25	1.385 (3)
C4—H4	0.9500	C21—C22	1.394 (3)
C5—C6	1.399 (3)	C21—H21	0.9500
C5—H5	0.9500	C22—C23	1.401 (3)
C6—C7	1.458 (3)	C22—H22	0.9500
C8—C9	1.524 (3)	C23—C24	1.402 (3)
C8—H8A	0.9900	C23—H23	0.9500
C8—H8B	0.9900	C24—C25	1.381 (3)
C9—C10	1.510 (3)	C24—H24	0.9500
C9—H9A	0.9900	C25—C26	1.491 (3)
			
C16—S1—C17	100.25 (10)	C11—C12—H12	119.8
C19—N3—C26	112.02 (17)	C12—C13—C14	119.1 (2)
C19—N3—C18	124.36 (17)	C12—C13—H13	120.5
C26—N3—C18	123.46 (18)	C14—C13—H13	120.5
C16—N1—C1	117.35 (18)	C13—C14—C15	120.5 (2)
C16—N2—C7	121.09 (18)	C13—C14—H14	119.7
C16—N2—C8	122.24 (17)	C15—C14—H14	119.7
C7—N2—C8	116.61 (17)	C14—C15—C10	121.03 (19)
N1—C1—C6	122.29 (19)	C14—C15—H15	119.5
N1—C1—C2	118.41 (18)	C10—C15—H15	119.5
C6—C1—C2	119.28 (19)	N1—C16—N2	124.87 (18)
C3—C2—C1	119.5 (2)	N1—C16—S1	119.56 (16)
C3—C2—H2	120.2	N2—C16—S1	115.56 (15)
C1—C2—H2	120.2	C18—C17—S1	111.35 (15)
C2—C3—C4	121.0 (2)	C18—C17—H17A	109.4
C2—C3—H3	119.5	S1—C17—H17A	109.4
C4—C3—H3	119.5	C18—C17—H17B	109.4
C5—C4—C3	119.5 (2)	S1—C17—H17B	109.4
C5—C4—H4	120.3	H17A—C17—H17B	108.0
C3—C4—H4	120.3	N3—C18—C17	111.64 (17)
C4—C5—C6	120.3 (2)	N3—C18—H18A	109.3
C4—C5—H5	119.9	C17—C18—H18A	109.3
C6—C5—H5	119.9	N3—C18—H18B	109.3
C1—C6—C5	120.4 (2)	C17—C18—H18B	109.3
C1—C6—C7	119.29 (19)	H18A—C18—H18B	108.0
C5—C6—C7	120.27 (19)	O2—C19—N3	124.9 (2)
O1—C7—N2	120.6 (2)	O2—C19—C20	129.5 (2)
O1—C7—C6	124.7 (2)	N3—C19—C20	105.56 (17)
N2—C7—C6	114.63 (18)	C21—C20—C25	121.8 (2)
N2—C8—C9	112.36 (17)	C21—C20—C19	129.78 (19)
N2—C8—H8A	109.1	C25—C20—C19	108.38 (18)
C9—C8—H8A	109.1	C20—C21—C22	117.0 (2)
N2—C8—H8B	109.1	C20—C21—H21	121.5
C9—C8—H8B	109.1	C22—C21—H21	121.5
H8A—C8—H8B	107.9	C21—C22—C23	121.4 (2)
C10—C9—C8	113.56 (17)	C21—C22—H22	119.3
C10—C9—H9A	108.9	C23—C22—H22	119.3
C8—C9—H9A	108.9	C22—C23—C24	120.8 (2)
C10—C9—H9B	108.9	C22—C23—H23	119.6
C8—C9—H9B	108.9	C24—C23—H23	119.6
H9A—C9—H9B	107.7	C25—C24—C23	116.9 (2)
C15—C10—C11	117.5 (2)	C25—C24—H24	121.6
C15—C10—C9	123.52 (18)	C23—C24—H24	121.6
C11—C10—C9	118.96 (19)	C24—C25—C20	122.1 (2)
C12—C11—C10	121.5 (2)	C24—C25—C26	129.8 (2)
C12—C11—H11	119.3	C20—C25—C26	108.15 (18)
C10—C11—H11	119.3	O3—C26—N3	124.6 (2)
C13—C12—C11	120.4 (2)	O3—C26—C25	129.6 (2)
C13—C12—H12	119.8	N3—C26—C25	105.84 (17)
			
C16—N1—C1—C6	3.7 (3)	C8—N2—C16—N1	172.78 (19)
C16—N1—C1—C2	−177.81 (18)	C7—N2—C16—S1	174.78 (14)
N1—C1—C2—C3	−176.32 (18)	C8—N2—C16—S1	−8.0 (2)
C6—C1—C2—C3	2.2 (3)	C17—S1—C16—N1	−6.58 (19)
C1—C2—C3—C4	0.3 (3)	C17—S1—C16—N2	174.18 (15)
C2—C3—C4—C5	−1.6 (3)	C16—S1—C17—C18	−75.70 (16)
C3—C4—C5—C6	0.3 (3)	C19—N3—C18—C17	−103.6 (2)
N1—C1—C6—C5	174.95 (18)	C26—N3—C18—C17	81.3 (2)
C2—C1—C6—C5	−3.5 (3)	S1—C17—C18—N3	−175.35 (14)
N1—C1—C6—C7	−7.8 (3)	C26—N3—C19—O2	176.6 (2)
C2—C1—C6—C7	173.73 (18)	C18—N3—C19—O2	1.1 (3)
C4—C5—C6—C1	2.3 (3)	C26—N3—C19—C20	−2.2 (2)
C4—C5—C6—C7	−174.93 (19)	C18—N3—C19—C20	−177.75 (17)
C16—N2—C7—O1	178.66 (18)	O2—C19—C20—C21	3.7 (4)
C8—N2—C7—O1	1.3 (3)	N3—C19—C20—C21	−177.6 (2)
C16—N2—C7—C6	0.1 (3)	O2—C19—C20—C25	−177.4 (2)
C8—N2—C7—C6	−177.24 (16)	N3—C19—C20—C25	1.3 (2)
C1—C6—C7—O1	−172.93 (19)	C25—C20—C21—C22	−0.5 (3)
C5—C6—C7—O1	4.3 (3)	C19—C20—C21—C22	178.3 (2)
C1—C6—C7—N2	5.6 (3)	C20—C21—C22—C23	0.9 (3)
C5—C6—C7—N2	−177.19 (18)	C21—C22—C23—C24	−0.6 (3)
C16—N2—C8—C9	97.7 (2)	C22—C23—C24—C25	−0.1 (3)
C7—N2—C8—C9	−85.0 (2)	C23—C24—C25—C20	0.6 (3)
N2—C8—C9—C10	176.31 (17)	C23—C24—C25—C26	−178.4 (2)
C8—C9—C10—C15	11.0 (3)	C21—C20—C25—C24	−0.3 (3)
C8—C9—C10—C11	−168.71 (19)	C19—C20—C25—C24	−179.33 (18)
C15—C10—C11—C12	0.5 (3)	C21—C20—C25—C26	178.95 (18)
C9—C10—C11—C12	−179.8 (2)	C19—C20—C25—C26	−0.1 (2)
C10—C11—C12—C13	0.2 (3)	C19—N3—C26—O3	−177.16 (19)
C11—C12—C13—C14	−0.5 (3)	C18—N3—C26—O3	−1.5 (3)
C12—C13—C14—C15	0.2 (3)	C19—N3—C26—C25	2.1 (2)
C13—C14—C15—C10	0.5 (3)	C18—N3—C26—C25	177.73 (17)
C11—C10—C15—C14	−0.8 (3)	C24—C25—C26—O3	−2.8 (4)
C9—C10—C15—C14	179.5 (2)	C20—C25—C26—O3	178.1 (2)
C1—N1—C16—N2	2.5 (3)	C24—C25—C26—N3	178.0 (2)
C1—N1—C16—S1	−176.67 (14)	C20—C25—C26—N3	−1.2 (2)
C7—N2—C16—N1	−4.4 (3)		

### Hydrogen-bond geometry (Å, º)

**Table:**

*D*—H···*A*	*D*—H	H···*A*	*D*···*A*	*D*—H···*A*
C13—H13···O1^i^	0.95	2.56	3.218 (3)	127
C17—H17*B*···O3^ii^	0.99	2.42	3.120 (2)	128
C21—H21···O2^iii^	0.95	2.45	3.346 (3)	157

Symmetry codes: (i) −*x*+3, −*y*+2, −*z*+2; (ii) −*x*+1, −*y*+1, −*z*+1; (iii) −*x*, −*y*+2, −*z*+1.
